# Supplementary material for: Bidirectional Phase Sensitivity in Holographic Phototransient Microscopy
Source: ACS Photonics. 2026 Oct 15;13(21):6460–6. doi: 10.1021/acsphotonics.6c01684 (PMC13742288; doi:10.1021/acsphotonics.6c01684)
Supplement: Supplementary file 1 [file ph6c01684_si_001.pdf]

Supplementary Information:

Bidirectional phase sensitivity in holographic phototransient microscopy

Emmanuel Kotu Robertson[1], Dennis van de Lockand[1], Matz Liebel[1,*]

[1] Department of Physics and Astronomy, Vrije Universiteit Amsterdam, De Boelelaan 1100, Amsterdam 1081 HZ, The Netherlands

[*] corresponding author: m.liebel@vu.nl

Supplementary information 1 – Plane wave recovery after back-reflection and transmission through substrate

To showcase that the imprinted phase profile acquired during the first pass through the sample rapidly disappears, we simulated a 5 µm PS (n=1.59) bead in air as a pure, flat, phase object with a spherical phase profile according to its z-projected thickness (peak phase retardation of 36 rad at an illumination wavelength of 515 nm). We then used the angular spectrum method to propagate the resulting field through CaF_2 with a thickness of 340 µm and 2000 µm, representing a “typical coverglass” and the substrate thickness used in our work (two times 1000 µm), respectively. Figure S1 shows that an essentially flat wavefront is recovered after 2000 µm with only a minor residual phase imprint being present after 340 µm. Importantly, even the 340 µm phase imprint is smaller than most “phase imperfections” in typical “plane wave” illumination sources as dust, speckle etc. generally impact the illumination wave.

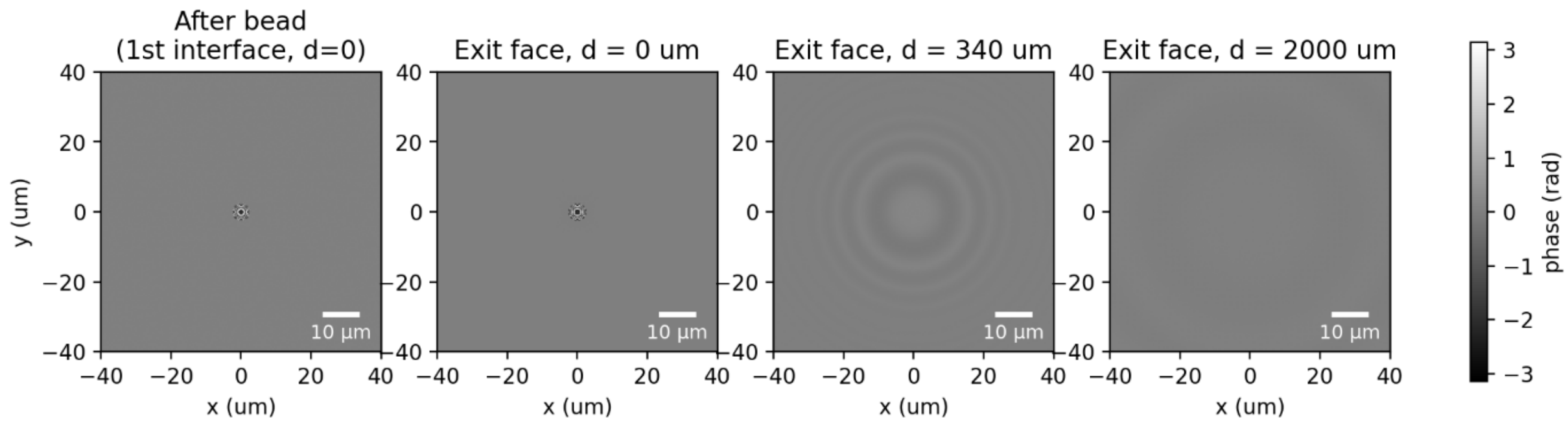

Figure S1. Transverse phase (wrapped, radians) immediately after the bead (left) and at the substrate exit face for d = 0, 340, and 2000 µm. The concentric-ring pattern imprinted by the bead spreads and dilutes with increasing thickness; by d = 2000 µm the field within the plotted window (±40 µm) is visually uniform.

Supplementary information 2 - Coherence gating characterization

To validate the path selectivity of the temporal coherence gate, we recorded the reconstructed interference amplitude versus reference-arm delay across the sample chamber depth (Figure S2A). Five peaks are resolved and assigned to the near-interface BWS field (B), the IFS channel generated by back-reflection at the distal surface of the CaF_2 substrate (A), the front and back

surfaces of the coverglass (C, D), and an additional weak reflection (E). Peak-to-peak spacings closely track the expected optical path lengths for each interface: the A–B separation (1.549 mm) is consistent with a double pass through the 1.06-mm-thick CaF_2 substrate (n = 1.433 at 515 nm; expected 1.519 mm, ~2% agreement), confirming the assignment of peak A to the distal-surface-reflected IFS field. The B–C spacing (0.197 mm) matches the ≈0.2 mm air gap between the substrate and coverglass, and the C–D spacing (0.269 mm) matches a double pass through the 0.17-mm-thick coverglass (n ≈ 1.58 at 515 nm). All measured peak widths (FWHM: A = 0.062 mm, B = 0.060 mm, C = 0.070 mm, D = 0.080 mm, E = 0.115 mm) are comparable to the coherence length expected from the 515 nm source bandwidth ($\Delta\lambda \approx 3$ nm, $L_c \approx \lambda^2/\Delta\lambda \approx 88$ μm), and are much narrower than the ≥0.2 mm separation between any two adjacent peaks. The IFS and BWS channels are therefore well resolved by the coherence gate, with negligible cross-talk between them.

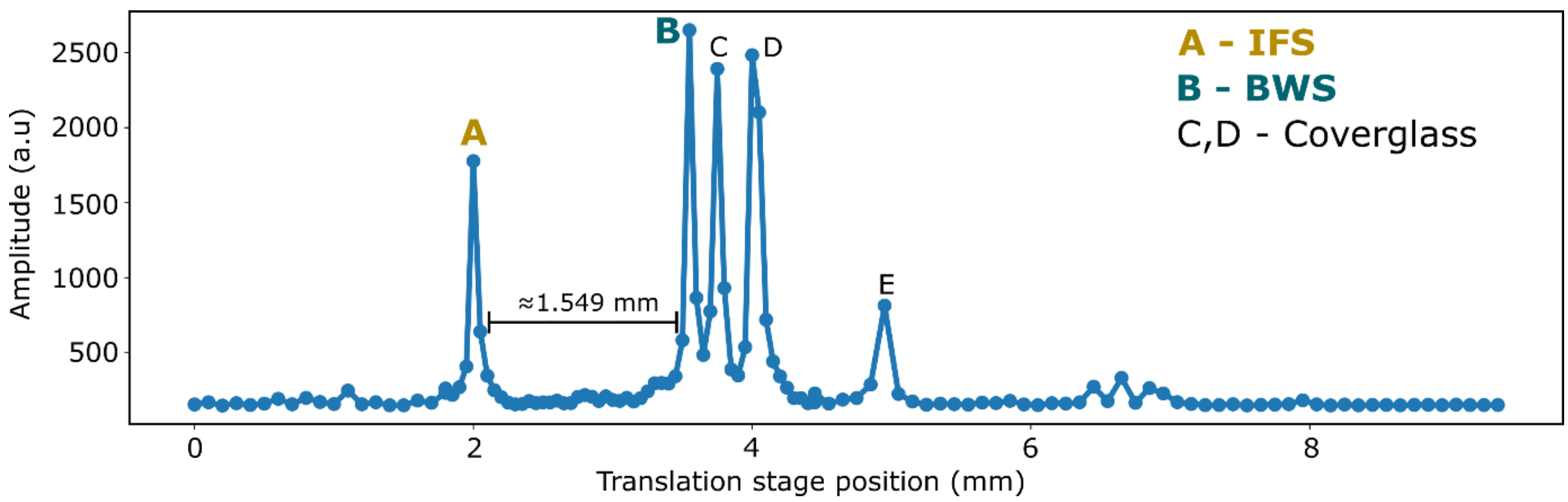

Figure S2A. Reference delay scan in BWS geometry.

As a control for parasitic reflections independent of the sample interfaces, we performed the same delay scan in the forward-scattering (FWS) configuration (Figure S2B). A single dominant FWS peak is observed (FWHM = 0.062), along with one weak secondary peak at a delay matching peak E in Figure S2A. As this feature appears in both configurations at the same delay and is well separated from the FWS, IFS, and BWS peaks used for quantitative analysis, we attribute it to a parasitic reflection from a microscope optic (e.g., the objective) rather than to the sample chamber or particle, confirming it does not contaminate the channels of interest.

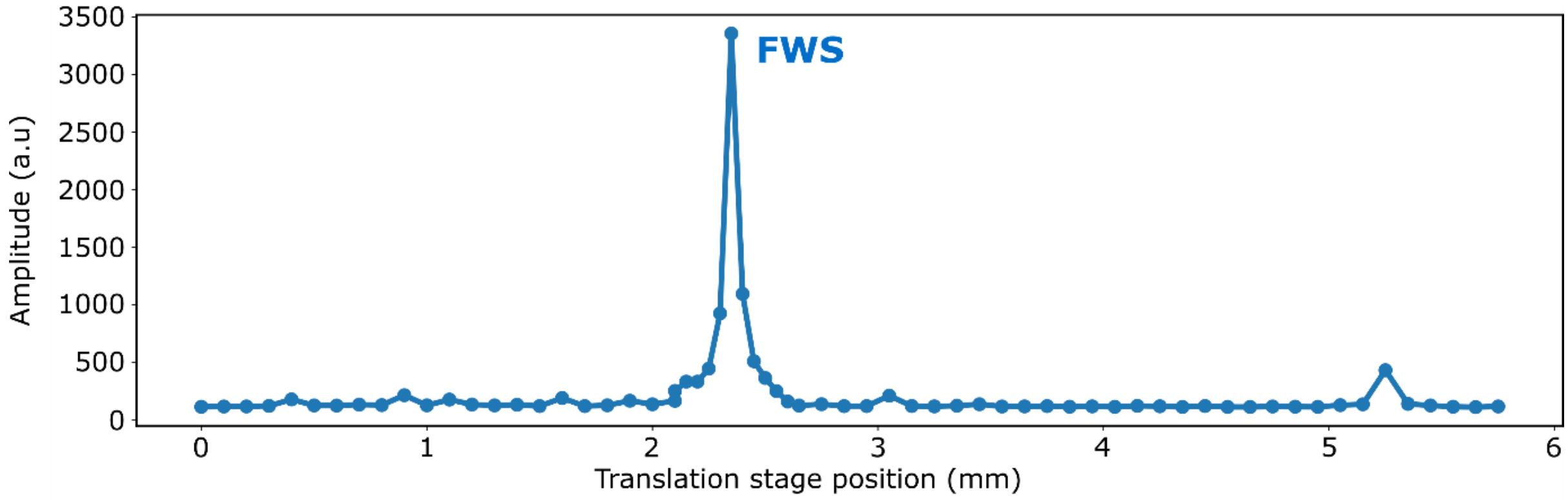

Figure S2B. Reference delay scan in FWS geometry.

Supplementary information 3 - Quantitative comparison of static bead phase across FWS, BWS and IFS

We imaged many individual 1 μm diameter polystyrene spheres on glass and propagated their fields via the angular spectrum method[1] to obtain through-focus signal evolution, indirectly accessing their Mie-scattering profile. Comparing this evolution (rather than absolute z-focus) also eliminates variations from shifting absolute focal positions between images. As shown in Figure S3 (mean ± std across all imaged spheres), FWS and IFS profiles are essentially identical, while BWS diverges significantly. The minor FWS/IFS differences likely stem from small misalignments in illumination k-vectors — e.g., a slight tilt relative to normal incidence (0°) in one of the two.

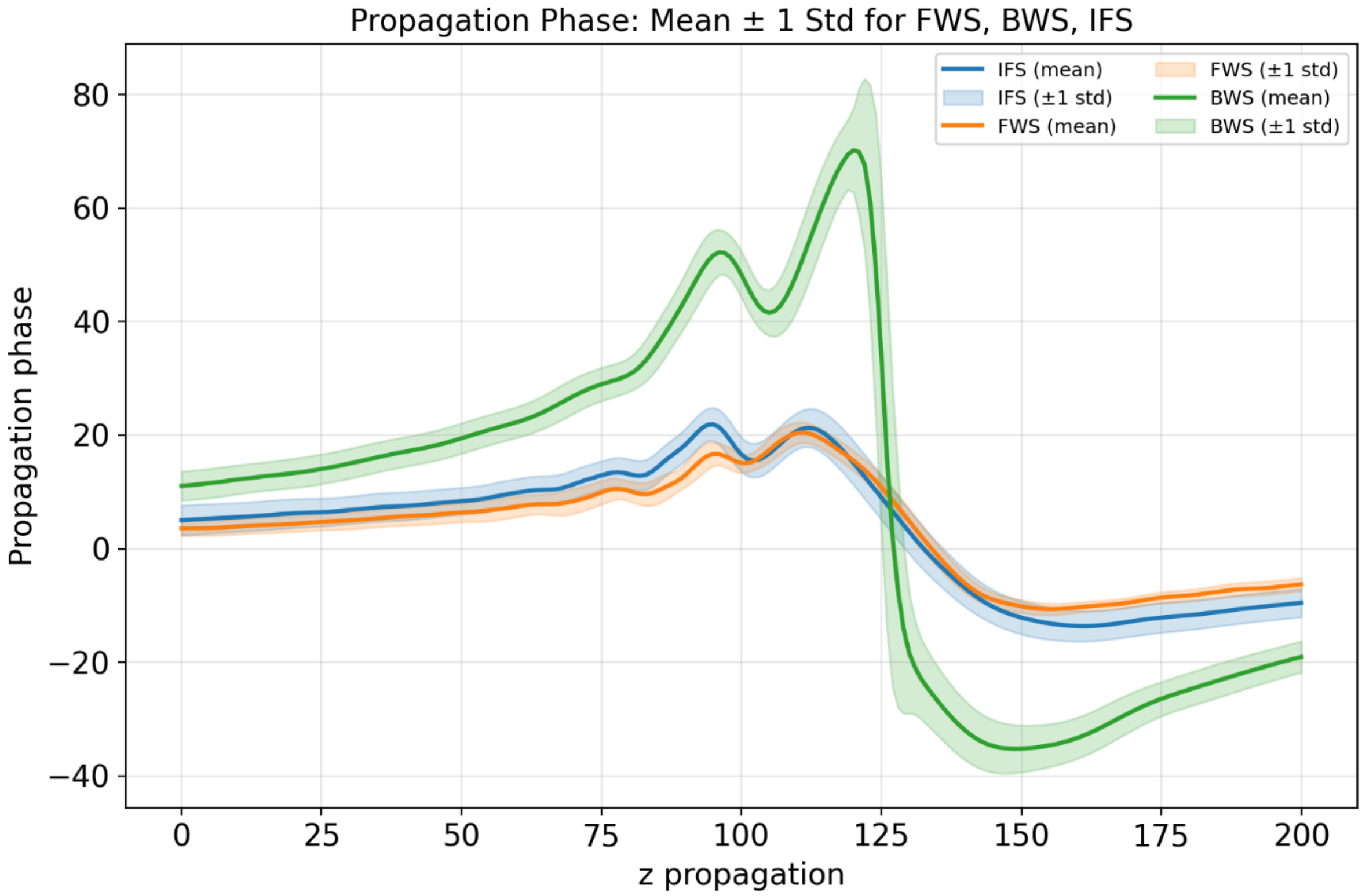

Figure S3. Quantitative comparison of IFS, FWS, and BWS signals, and focus-independence check.

REFERENCES

1. Ortega Arroyo, J. & Liebel, M. The Next Dimension: Digital Holography for 3D Interferometric Scattering. *ACS Nano* **19**, 38630–38640 (2025).
